# Septic shock due to community-acquired *Pseudomonas aeruginosa* necrotizing fasciitis: A case report and literature review

**DOI:** 10.3892/etm.2014.1628

**Published:** 2014-03-21

**Authors:** GUANG-JU ZHAO, GUANG-LIANG HONG, JIE-QUAN LIU, YANG LU, ZHONG-QIU LU

**Affiliations:** Emergency Department, The First Affiliated Hospital of Wenzhou Medical University, Wenzhou, Zhejiang 325000, P.R. China

**Keywords:** necrotizing fasciitis, *pseudomonas aeruginosa*, septic shock

## Abstract

Necrotizing fasciitis is a rare but fatal infection, characterized by the rapid progression of necrosis of the fascia, skin, soft tissue and muscle. The most common bacteria associated with necrotizing fasciitis is group A *streptococcus*, although other pathogens have also been implicated. In the present study, a case of community-acquired necrotizing fasciitis, complicated with septic shock and multiple organ dysfunction syndromes due to *Pseudomonas aeruginosa*, is presented. Despite intensive medical treatment, the condition of the patient deteriorated rapidly and the patient subsequently succumbed to multiple organ failure. In view of the rapid progression and high mortality rate of this disease, early surgery, as well as novel therapeutic approaches for septic shock are required to improve the outcome for patients.

## Introduction

Necrotizing fasciitis is a rare but fatal infection, characterized by the rapid progression of necrosis of the fascia, skin, soft tissue and muscle. The most common bacteria associated with necrotizing fasciitis is group A *streptococcus*, however, other pathogens, including *Clostridium*, *Vibrio vulnificus* and *Bacteroides fragilis* have been implicated (1). *Pseudomonas* is a rare pathogen that causes necrotizing fasciitis (2). In the present study, a case of community-acquired necrotizing fasciitis with septic shock due to *Pseudomonas aeruginosa* is presented, as well as a review of the literature on the current therapeutic strategies for this condition.

## Case report

A 65-year-old male was admitted to the Emergency department of The First Affiliated Hospital of Wenzhou Medical University (Wenzhou, Zhejiang, China) due to a one-day history of fever and pain in his left leg. The patient had a history of myeloma approximately one year prior to admission, which was treated with four cycles of chemotherapy. This was followed by oral medication therapy (melphalan, dexamethasone and thalidomide). In addition, the patient had a history of type 2 diabetes mellitus for two years and hypertension for three years. The patient had no history of leg ulcers or direct trauma to the lower limbs prior to admission.

**Figure 1:**
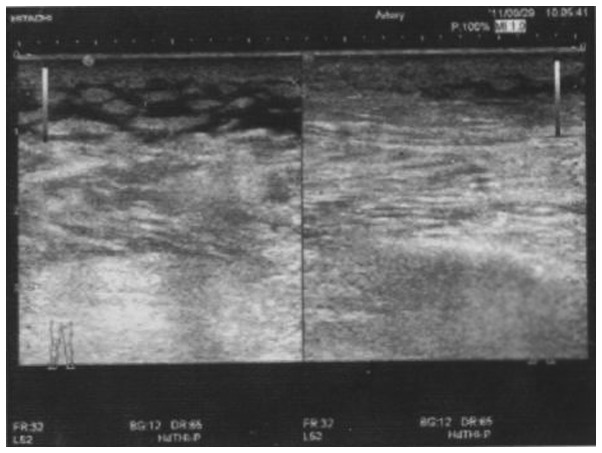
Ultrasonography revealed subcutaneous edema of the lower limbs, which was more marked in the left limb. No gas formation was observed in either leg and deep vein thrombosis was also not observed.

On the day of admission, at 6:00 am, the patient was febrile with a temperature of 39.4°C and presented with hypertension (70–80/40–50 mmHg). Physical examination revealed swelling and pyrexia of the left lower limb. Initial blood investigations revealed a decrease in white cell count of 1.18×10^9^/l and elevated levels of C-reactive protein (76.70 mg/l). The arterial blood gas values were as follows: pH 7.24; PaO_2_, 108.2 mmHg; PaCO_2_, 18.9 mmHg; HCO_3_, −19.6 mmol/l; and base excess (BE), −12.3 mmol/l. The level of creatine kinase (CK) was markedly increased (1208 U/l), while the CK-MB level was only slightly increased (26 U/l) and the level of troponin T was normal (<0.01 μg/l), suggesting the presence of rhabdomyolysis. Ultrasonography revealed subcutaneous edema of the lower limbs, which was more marked in the left limb (Fig. 1). No gas formation was observed in either leg and deep vein thrombosis was also not observed. The clinical characteristics of the patient indicated lower limb necrotizing fasciitis (stage 3) and bacterial septic shock. Early fluid resuscitation was initiated and norepinephrine was administered intravenously according to the central venous pressure and mean arterial pressure. Blood and bullous fluid samples from the affected skin were obtained, and empiric antimicrobial therapy (piperacillin sodium/tazobactam sodium and linezolid) was initiated. Due to the marked neutropenia, a complete workup for immunodeficiency was performed in association with the empirical use of human immunoglobulin and recombinant human granulocyte colony-stimulating factor (rhG-CSF).

**Figure 2:**
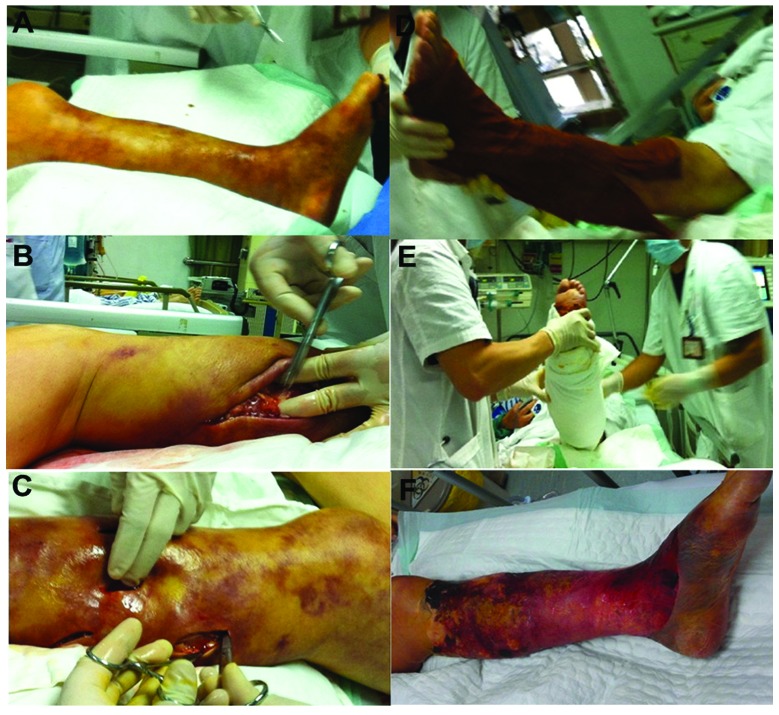
Emergency incision and drainage of the lower left limb of a patient with necrotizing fasciitis. (A) Left lower limb infection with *Pseudomonas aeruginosa*; (B) single large incision; (C) multiple small incisions; (D) wet compress with 2.5% iodophor, during the emergency incision and drainage; (E) simple bandaging post emergency incision and drainage; (F) additional debridement once the hemodynamics returned to normal.

The patient was admitted to the emergency intensive care unit (EICU) at 15:00 and was administered norepinephrine intravenously. However, the patient was not able to maintain hemodynamic stability. The respiratory rate of the patient was 40–60 breaths/min and his heart rate ranged between 130 and 150 beats/min. The arterial blood gas values were as follows: pH 7.040; PaO_2_, 48.5 mmHg; PaCO_2_, 72.5 mmHg; HCO_3_, −19.6 mmol/l; and BE, −12.3 mmol/l. Endotracheal intubation was performed and mechanical ventilation was established. An emergency bedside echocardiology was obtained to exclude heart disease. Emergency incision and drainage was then performed on the patient. Subcutaneous vein thrombosis, subcutaneous tissue necrosis as well as muscle and full-thickness necrosis were observed during the surgery. Necrotizing fasciitis was confirmed by exploration and pathological examination, and fluid aspirate was sent for immediate culture. Continuous renal replacement therapy was then initiated. When the hemodynamics of the patient returned to normal, an additional debridement was performed. All infected and necrotic skin as well as subcutaneous tissue were radically excised up to the bleeding healthy edges (Fig. 2).

However, despite the treatments mentioned above, the clinical condition of the patient did not improve. The platelet count of the patient continued to decline and coagulation disorders were not able to be modified. At this time, linezolid was replaced with teicoplanin. *P. aeruginosa* was isolated from the blood culture and bullous fluid of the affected tissue four days after admission, however, no anaerobe was isolated. Based on the susceptibility test results, treatment with teicoplanin was discontinued and piperacillin/tazobactam sodium treatment was continued as definitive therapy. Following 10 days in EICU, the patient suddenly lost consciousness. Cyanosis was observed, as well as mydriasis and the papillary light reflex was lost. The heart rate of the patient decreased to 30–40 beats/min and cardiopulmonary resuscitation was immediately initiated. However, the patient deteriorated rapidly and succumbed to multiple organ failure. Written informed consent was obtained from the patient for publication of this case report and any accompanying images.

## Discussion

Necrotizing fasciitis and sepsis caused by *P. aeruginosa* is a rare but life-threatening disease, which occurs more frequently in patients suffering from alcoholism, diabetes or immunocompromised conditions (3). In the present case study, the patient presented with neutropenia, which was possibly secondary to the immunosuppressive medication and septic shock. In addition, the patient had diabetes mellitus, which may also lead to immune system dysfunction. Stopping the administration of immunosuppressive drugs and administering rhG-CSF in neutropenic patients may improve clinical outcomes (4). In the present case, the neutrophil count returned to normal after treatment with G-CSF for 4 days. However, no marked clinical improvement was observed, which may be due to the rapid progression of the disease complicated with septic shock and multiple organ dysfunction syndromes (MODS).

The serious complications of necrotizing fasciitis, including septic shock and MODS, require fluid resuscitation, antimicrobial therapy and early aggressive surgical debridement (3). The initial hemodynamic resuscitation of the patient was performed according to early goal-directed therapy (EGDT), which has been demonstrated to decrease mortality in patients with severe sepsis and septic shock (5,6). In addition, previous studies have demonstrated that the administration of appropriate antibiotics is one of the various early resuscitation interventions that need to be performed within 1 h of qualification of EGDT in patients with critical infections (5,6). Therefore, the patient received initial combination therapy of piperacillin/tazobactam sodium and linezolid immediately following collection of a blood sample and bullous fluid from the affected skin. When thrombocytopenia was detected, teicoplanin was administered instead of linezolid. Following completion of the antimicrobial susceptibility test, teicoplanin treatment was discontinued and piperacillin/tazobactam sodium was continued as the definitive therapy. Cefoperazone/sulbactam sodium was not selected due to the potential to cause coagulation disorders.

Continuous renal replacement therapy (CRRT) was performed when the patient reached state 2 AKI according to Acute Kidney Injury Network (AKIN) criteria. Several experimental and clinical studies have previously reported that blood purification therapies are effective in restoring immune function by clearing inflammatory mediators from the plasma, as well as improving physiological parameters, including hemodynamics and oxygenation (7). CRRT is the preferred mode of renal replacement in sepsis-induced acute kidney injury (AKI). The data from the acute tubular necrosis and renal studies indicated that vasopressor-dependent ICU patients on CRRT evolve to chronic dialysis less frequently compared with patients who receive intermittent therapies (8). Furthermore, CRRT is recommended during the acute phase of AKI, particularly in patients with severe hemodynamic instability or when extensive fluid removal may allow more effective drug therapy (8). However, it must be taken into account that numerous antibiotics are largely removed by continuous renal replacement therapies (9). In the present case study, the pharmacokinetic characteristics of piperacillin/tazobactam were not measured. However, the adequacy of piperacillin/tazobactam 4.0/0.5 every 8 h was reported in a study of nine patients on CRRT who maintained concentrations >125 μg/ml for the whole time interval (10).

Previous studies have demonstrated that prompt and radical surgical debridement is an effective treatment for necrotizing fasciitis. Delaying surgery by 24 h increased the mortality rate associated with necrotizing soft tissue infections from 35 to 53%, with 100% mortality if surgery is not performed within three days (11). The ultimate outcome of the patient also depends on the completeness of surgical debridement (12); the excision margin should be healthy bleeding tissue with a normal appearance (11,12). However, our previous study demonstrated that, compared with radical surgical debridement, incision and drainage was also effective and safe, particularly in patients with septic shock complicated with MODS and severe coagulation disorders (13). Additionally, this type of surgical approach is simple, quick and results in little blood loss (13). Therefore, in the present case study, incisions and drainage of the involved extremities were performed. Selective debridement was then performed once the hemodynamics of the patient had stabilized. However, the patient succumbed to sepsis-induced MODS.

In conclusion, in the present study, a case of community-acquired *P. aeruginosa* necrotizing fasciitis complicated with septic shock and MODS was reported. Due to the rapid progression and high mortality rate of the disease, early surgery, as well as novel therapeutic approaches for septic shock, are required to improve the outcome of patients.
